# Tunable 3D Hydrogel Microchannel Networks to Study Confined Mammalian Cell Migration

**DOI:** 10.1002/adhm.202100625

**Published:** 2021-10-20

**Authors:** Katharina Siemsen, Sunil Rajput, Florian Rasch, Fereydoon Taheri, Rainer Adelung, Jan Lammerding, Christine Selhuber‐Unkel

**Affiliations:** ^1^ Institute for Materials Science Kiel University Kiel D‐24143 Germany; ^2^ Institute for Molecular Systems Engineering (IMSE) Heidelberg University Heidelberg 69120 Germany; ^3^ Meinig School of Biomedical Engineering & Weill Institute for Cell and Molecular Biology Cornell University Ithaca NY 14853 USA; ^4^ Max Planck School Matter to Life Jahnstraße 29 Heidelberg 69120 Germany

**Keywords:** 3D architecture, cell migration, confined microarchitecture, hydrogels, nuclear envelopes

## Abstract

Cells adapt and move due to chemical, physical, and mechanical cues from their microenvironment. It is therefore important to create materials that mimic human tissue physiology by surface chemistry, architecture, and dimensionality to control cells in biomedical settings. The impact of the environmental architecture is particularly relevant in the context of cancer cell metastasis, where cells migrate through small constrictions in their microenvironment to invade surrounding tissues. Here, a synthetic hydrogel scaffold with an interconnected, random, 3D microchannel network is presented that is functionalized with collagen to promote cell adhesion. It is shown that cancer cells can invade such scaffolds within days, and both the microarchitecture and stiffness of the hydrogel modulate cell invasion and nuclear dynamics of the cells. Specifically, it is found that cell migration through the microchannels is a function of hydrogel stiffness. In addition to this, it is shown that the hydrogel stiffness and confinement, influence the occurrence of nuclear envelope ruptures of cells. The tunable hydrogel microarchitecture and stiffness thus provide a novel tool to investigate cancer cell invasion as a function of the 3D microenvironment. Furthermore, the material provides a promising strategy to control cell positioning, migration, and cellular function in biological applications, such as tissue engineering.

## Graphical abstract

The tunable hydrogel microarchitecture and stiffness in microchannel networks provide a novel strategy to investigate cancer cell invasion. For example, cell invasion, nuclear dynamics, cell migration, and nuclear envelope ruptures are controlled by the structural and mechanical properties of the microenvironment.

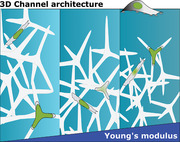

## 1 Introduction

Cell migration is essential for proper immune responses, wound repair, and tissue homeostasis in adults.^[^ ^1^ ^]^ For a cell to migrate through constrictions smaller than the size of the cell, which is frequently observed within interstitial tissue spaces and basement membranes, it is essential that either the cell or its surrounding microenvironment can be sufficiently deformed.^[^ ^2^, ^3^, ^4^ ^]^ Cell migration plays a crucial role during cancer metastasis, i.e., the spreading of cancer cells from the primary tumor to surrounding tissues and distant organs, with cells migrating in a single or collective form.^[^ ^5^, ^6^ ^]^ Furthermore, as external forces and the mechanics of the extracellular environment play a large role in cellular processes, understanding how cells migrate through tight spaces without damage is essential.

The influence of cell migration as a function of confinement is often investigated with synthetic quasi‐3D structures prepared by microstructured polydimethylsiloxane (PDMS).^[^ ^7^, ^8^, ^9^, ^10^, ^11^ ^]^ The effect of the microstructures on cell migration strongly depends on cell and tissue type.^[^ ^6^ ^]^ Both confinement as well as topographical aspects have been discussed for controlling cell migration,^[^ ^12^ ^]^ and it is well known that different aspects, including cell adhesiveness and contractility, alter cell migration through the microstructured space.^[^ ^13^ ^]^

A recently investigated phenomenon is how the cell, and in particular the nucleus, behaves and travels within confined environments.^[^ ^14^, ^15^ ^]^ Such travels through micron‐sized constrictions can also affect the integrity of the nuclear envelope (NE), which separates the nuclear content from the cytoplasm.^[^ ^16^, ^17^ ^]^ The NE acts as a selective barrier to protect the genome, and it enables processes such as DNA duplication as well as transcription.^[^ ^18^, ^19^ ^]^ A number of studies have explored the migration of cells through constrictions, which report substantial deformation of the nucleus, resulting in rupture of the NE and DNA damage;^[^ ^10^, ^20^, ^21^, ^22^ ^]^ as well as instances where mechanical stress alters, for example, stem cell differentiation.^[^ ^23^ ^]^

To develop a better understanding of how cells migrate in different environments, it is essential to synthesize materials that mimic the matrix geometry present within human tissues, ideally also representing the soft, 3D nature of the extracellular environment. It is undeniable that the extracellular matrix (ECM) is viscoelastic in nature and can be modified by the cells, so that in some applications viscoelastic hydrogels have been used to mimic these environments by introducing dynamic bonds.^[^ ^24^ ^]^ In addition, collagen matrices of different stiffness are commonly used materials to test 3D cell migration and the impact of pore size and scaffold stiffness on NE integrity.^[^ ^10^, ^25^, ^26^ ^]^ One limitation of collagen matrices, however, is the challenge to independently control pore size and matrix stiffness, making systematic studies of the effect of extracellular microstructure and mechanics on cell migration difficult.

Additionally, collagen matrices exhibit a very heterogeneous distribution of pore sizes, presenting an additional challenge. Furthermore, to control porosity within these materials, methods such as salt leaching, gas foaming, microporous annealed particles, or phase separation can be used.^[^ ^27^ ^]^ As successful as these can be, creating highly complex, interconnected microchannel structures are beyond their remit.

Here, we present the development and application of novel 3D structured hydrogels with distinct microchannel architecture (via a sacrificial template model) to study mammalian cell migration in well‐defined geometries, and to visualize NE ruptures as cells migrate through the confined microchannels. As hydrogels share their hydrated and soft nature with the extracellular matrix (ECM), it makes them ideal candidates for studying their impact on cell migration.^[^ ^28^, ^29^, ^30^ ^]^ Importantly, our macroscopic, easy‐to‐fabricate materials exhibit an interconnected microchannel network allowing cell migration through the entire materials. The aim of this proof‐of‐concept study was to demonstrate that the 3D hydrogel scaffolds, in which microchannel architecture and matrix stiffness can be tuned independently, allows for systematically studying cell migration, nuclear motion and NE rupture, and thus to demonstrate the potential of this material for a broad range of biomedical applications.

## 2 Experimental Section

### 2.1 Construction of 3D Microstructured Hydrogel Matrices with Interconnected Channels

The interconnected channel architecture inside the hydrogel was created by a sacrificial template synthesis as reported previously in literature.^[^ ^31^ ^]^ The sacrificial template was prepared from PVB tetrapodal ZnO (t‐ZnO) powder.^[^ ^30^ ^]^ The PVB t‐ZnO was synthesized via flame transport synthesis, with PVB as sacrificial polymer and tetrapodal arm diameter ranging from 1 to 8 µm.^[^ ^30^ ^]^ Next, t‐ZnO was pressed into a mold to create templates of desired size and t‐ZnO density. The t‐ZnO density was chosen to be 0.9 gcm^−3^. To form the interconnected channel architecture, the t‐ZnO templates were sintered at 1150 °C for 5 h.

A predefined solution (**Table** **1**) of pAAm was poured over the ZnO templates. These solutions were adjusted on crosslinker concentration. Acrylamide (40% solution, Bio‐Rad), and 4‐(2‐hydroxyethyl)‐1‐piperazineethanesulfonic acid buffer (HEPES, pH 8,5, Sigma Aldrich) were added to a beaker. To this, depending on desired hydrogel mechanical properties, 1vol.%, 4vol.% or 16vol.% of *N*,*N′*‐methylenebisacrylamide (Bio‐Rad) was added. Acrylic acid *N‐*hydroxysuccinimide ester (A‐NHS, 0.003 g, Sigma Aldrich) was suspended in dd.H_2_O (200 µl, AppliChem) in a beaker. The pH was adjusted using sodium hydroxide (2.5 M, Sigma Aldrich) in 1 µl increments to a value of 7. The volume was then made up to a total of 492.01 µl with dd.H_2_O (AppliChem) Ammonium persulfate (Sigma Aldrich) was then added to the mixture, and the solution was placed in a desiccator for 5 min to allow the solution to de‐gas. *N*,*N*,*N′*,*N′*‐tetramethylenediamine (Sigma‐Aldrich) was then added and mixed, bringing the total volume to 500 µL, resulting in the precursor solution.

**Table 1:** Components of hydrogel precursor solution

Volumetric crosslinker concentration	1 vol%	4 vol%	16 vol%
Acrylamide	94.69 µL		
(HEPES, pH 8,5)	5 µL		
*N*,*N′*‐methylenebisacrylamide	5 µL	20 µL	80 µL
Acrylic acid *N*‐hydroxysuccinimide ester	0.003 g		
Sodium hydroxide	To adjust pH to 7		
dd.H_2_O	Fill up to 492,01 µL		
Ammonium persulfate	7.5		
*N*,*N*,*N′*,*N′*‐Tetramethylenediamine	0.49 µL		
Total volume	500 µL		

This was then poured over the ZnO templates. After polymerization of the pAAm hydrogel, the t‐ZnO was dissolved via hydrolyzation in hydrochloric acid (0.5 m, Sigma‐Aldrich) and washed under agitation in dd.H_2_O water until a neutral pH was reached for the supernatant. The Young's modulus of each hydrogel was adjusted through using three different volumetric crosslinker concentrations.

### 2.2 Biofunctionalization of 3D Microstructured Hydrogel Matrices with Interconnected Channel Architecture

The hydrogel samples were immersed in ethanol (70 vol%, Walter) for 15 minutes and subsequently washed three times with HEPES (pH 8.5). Samples were then incubated in a collagen I (0.5 mg mL^−1^, AdvancedBiomatrix) solution at 4 °C overnight. Finally, the samples were washed three times with phosphate‐buffered saline (PBS, Sigma‐Aldrich) ready for cell experiments.

### 2.3 Visualization and Diameter Determination of 3D Microstructured Hydrogel Matrices with Interconnected Channel Architecture

pAAm hydrogels were dehydrated using an ethanol series (70‐99 vol%) for 15 minutes at each concentration. Samples were then immersed into a solution of Fluorescein isothiocyanate–Dextran 500.000–Conjugate (FITC–Dextran, 1.32 mg mL^−1^, Sigma‐Aldrich) over night. Using a spinning disc confocal microscope, samples placed in a glass bottom petri dish (IBIDI) were analyzed, the samples were analyzed through Z‐stacks with a step width of 1.99 µm. These were recorded for six different positions per sample and for all three different hydrogel compositions. The resultant *z*‐stacks were visualized using the voxel view intensity representation, rendered with the xCellence rt software (Olympus). The diameters of the channels were also measured with a measurement tool from this software.

### 2.4 Mechanical Properties of Hydrogels in Respect to their Crosslinker Concentration

For the mechanical properties of the hydrogels used, bulk samples with the different volumetric crosslinker concentrations were prepared from components in Table 1 in a 10 times higher quantity. Samples from 800 µL precursor solution were polymerized in a PTFE mold with a diameter of 1 cm and a height of 5 mm. Next, after polymerization hydrogel samples were immersed into dd.H_2_O for 2 weeks at room temperature. The investigation of the mechanical properties was conducted via indentation with a set‐up reported earlier.^[^ ^30^ ^]^ Hydrogel samples (*n* ≥ 4, three times per sample) were indented with a PTFE sphere with a diameter of 6 mm and a velocity of 0.1 mm s^−1^. The indentation depth was kept constant at 10% sample thickness.^[^ ^30^ ^]^

### 2.5 Cell Experiments

Fibrosarcoma cells (HT1080) stably expressing NLS‐GFP and H2B‐RFP^[^ ^16^ ^]^ were cultured in DMEM (Biochrom GmbH) containing 10% fetal bovine serum (FBS, Biochrom GmbH), and 1% penicillin streptomycin (Sigma‐Aldrich) under cell culture conditions (37 °C, incubator with 5% CO_2_). For the experiments, a 3D microstructured hydrogel matrix with interconnected channel architecture with 6 mm diameter and a maximum height of 2 mm was placed into a transwell (1 µm pore size, Millicell Cell culture Inserts, Merck). This transwell was then placed into a 24‐well plate (Sarstedt). Below the transwell, 2 mL of cell medium with FBS was filled into the well plate. On top of the sample inside the transwell 50 000 cells were seeded with 600 µL medium. After 2 h of incubation, the medium inside the transwell was replaced with FBS free medium. This set‐up was incubated for 5 days. 24 h before the experiments, the DMEM medium was exchanged to CO_2_ independent medium FluoroBrite DMEM (in ‚FlouroBrite‘ medium, Gibco) containing 10% FBS, 1% penstrep, 1/50 GlutaMAX (Gibco, Germany), and 1/40 2‐(4‐(2‐Hydroxyethyl)‐1‐piperazinyl)‐ethansulfic acid (HEPES, pH 8.5, Sigma). The medium inside the transwell was without FBS.

For imaging, samples were transferred to a homebuilt PDMS chamber (5 mm × 8 mm) attached to a petridish with glass bottom (IBIDI) and filled with "FlouroBrite" medium.

To provide suitable cell culture conditions a heating chamber (MI‐IBC, Olympus) was used during imaging.

### 2.6 Software and Statistics

Image acquisition was conducted through the Olympus xCellence imaging software for life science microscopy, utilizing Fiji (ImageJ) as a means of image analysis. GraphPad Prism was utilized for plotting 2D data and OriginLab: Origin was used for 3D scatter plots. Custom figures and schematics were designed using Inkscape. For each graph produced, the mean was calculated and error bars signify standard deviation. One‐way ANOVA (Kruskal‐Wallis test) was used for statistical analysis of corresponding plots using GraphPad Prism. Using an uncorrected Dunn's test, *p* values were compared as stand‐alone values (alpha 0.05).

## 3 Results and Discussion

### 3.1 Mimicking Elasticity of Soft Tissue and the ECM with Structured Polymer Hydrogels

**Figure 1:**
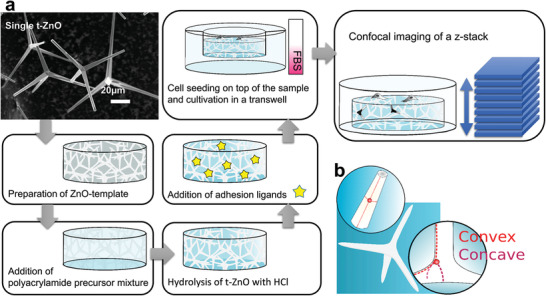
a) Process of assembling of microstructured hydrogels, starting with single zinc oxide tetrapod's (t‐ZnO) that are sintered into a network structure (ZnO‐template), which is embedded with a polyacrylamide hydrogel. After hydrolyzing the t‐ZnO structure, this results in a continuous microchannel network within the hydrogel. The hydrogel channels are biofunctionalized with extracellular matrix proteins or other adhesion ligands. Subsequently, the hydrogels are mounted on a transwell plate, a chemotactic gradient is applied to stimulate cell migration, and cell invasion is visualized by confocal imaging of cells within the hydrogel. b) Geometric patterning of channel and channel intersection within the synthesized hydrogel structure.

Polyacrylamide hydrogels (pAAm) are often used for studying cell‐materials interactions as a function of hydrogel stiffness.^[^ ^32^ ^]^ To explore their potential as a 3D structured material, we equipped them with an interconnected microchannel network. The microchannel network was successfully synthesized using a sacrificial template method based on the polymerization of a hydrogel around a microstructured zinc oxide (ZnO) template (**Figure** **1**).^[^ ^30^ ^]^ In detail, ZnO tetrapod's (t‐ZnO), were interconnected via a sintering process to create a unique large‐scale 3D network.^[^ ^32^, ^33^ ^]^ After dissolving the ZnO network, the inverse structure of the template is imprinted within the hydrogel, leading to interconnected microchannels throughout the hydrogel. Based on previous X‐ray microtomography measurements, the scaffolds have a connectivity >70%.^[^ ^34^ ^]^

To support cell–material interactions for anchor‐dependent cells, the pAAm hydrogels were cosynthesized with an acrylic acid derivative containing a *N‐*hydroxysuccinimide, which enables the conjugation of proteins.^[^ ^35^ ^]^ For the current studies, the hydrogel was biofunctionalized with collagen I to promote cell adhesion and migration of HT1080 fibrosarcoma cells. Covalent integration of collagen I resulted in the successful adhesion of cells to the hydrogel surface (Figure S1, Supporting Information), in line with previous reports.^[^ ^35^, ^36^ ^]^

**Figure 2:**
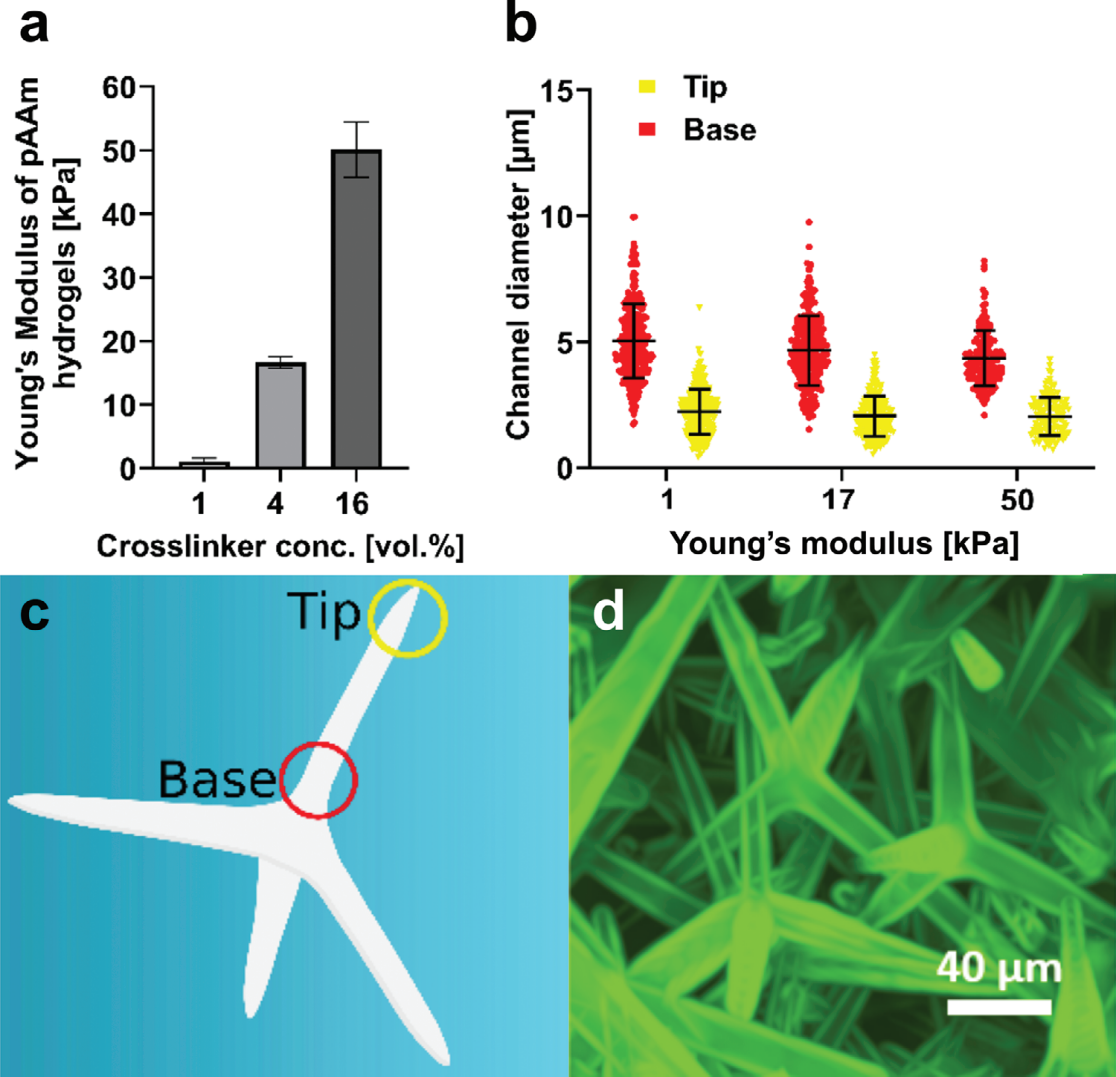
a) Indentation tests to determine Young's moduli of pAAm hydrogels at different crosslinker concentrations (number of samples tested (*N* ≥ 4) number of indents per sample (*n* = 3)) showing mean and standard deviation (SD). b) Base (red) and tip (yellow) diameter directly after preparation, measured through fluorescent imaging via spinning disk confocal microscopy for each hydrogel stiffness, showing mean ± standard error. c) Schematic of a single structure formed from a tetrapod. d) z‐stack fluorescent image (FITC‐Dextran) of interconnected architecture within the pAAm hydrogels.

A unique feature of our material is that the microchannel network can be formed independent of hydrogel stiffness, and that percolation of the microchannel network is reached even at low channel densities.^[^ ^30^ ^]^ Furthermore, hydrogels have macroscopic total dimensions, which is challenging to reach with other methods, such as direct laser writing.^[^ ^37^ ^]^ Hydrogels were further tailored with different crosslinker concentrations (*N*,*N′*‐methylenebisacrylamide). The crosslinker concentration‐dependent elasticity was determined via indentation tests, resulting in hydrogels with Young's moduli of 1, 17 and 50 kPa respectively (**Figure** **2**a). Utilizing a macro indentation method (approximately penetrating 10% of our sample thickness), we did not observe stiffening of our samples. These elastic moduli reflect a range of physiological conditions found in vivo.^[^ ^38^, ^39^, ^40^ ^]^

To characterize the microchannel network, we quantified the diameter of the microchannels in hydrogels made with different crosslinker concentrations (Figure 2b). For each material, channel diameter measurements were taken at the location corresponding to the tetrapod base and tip (Figure 2c). As expected, the tetrapod tip locations had smaller channel diameters than the base locations (Figure 2b), corresponding to the geometry of the individual and sintered tetrapods (Figure 2c,d). The variation in the microchannel diameters (within the range of about 1 to 10 µm) is a result of the stochastic nature related to the sintering process of the t‐ZnO Templates^[^ ^32^ ^]^ and is similar to the variation in pore size found in biological ECM.^[^ ^41^ ^]^ We observed a mild cross‐linker concentration‐dependent effect as previously described in literature.^[^ ^42^ ^]^ This is likely due to the swelling properties of hydrogels, which are adapted by the stoichiometric components such as the crosslinker concentration.

### 3.2 HT1080 Migrate through the Interconnected Channel Networks in 3D Hydrogels

To investigate the behavior of cells from soft tissue in a constricted 3D hydrogel microchannel microenvironment, we visualized migration of fibrosarcoma cells (HT1080) in the hydrogel by live‐cell microscopy. The HT1080 cells were genetically modified to stably express fluorescently labeled histone H2B (H2B‐RFP) and a reporter for NE rupture consisting of a green fluorescent protein with a nuclear localization sequence (NLS‐GFP). This strategy has recently successfully been used to study the effect of microchannels within PDMS substrates on NE rupture of MDA‐MB‐231 breast cancer cells and HT1080 cells.^[^ ^10^, ^22^ ^]^ Here, we explored the effects of the hydrogel microarchitecture (i.e., channel diameter) and elasticity on cell migration, localization within the microchannels, and nuclear structure and mechanics, including nuclear diameter, positioning, spatial dynamics, and NE rupture.

#### 3.2.1 Hydrogel Microarchitecture and Stiffness Control Cell Migration

Cell migration is highly dependent on both surface chemistry and material stiffness. As the microchannels in our 3D hydrogels consist of different interconnected shapes, it is possible to investigate whether specific channel geometries are preferentially occupied by the cells: microchannels, channel intersections, and the surface of the material. In this study, cell migration within the hydrogel is initially supported during cultivation with an 10% FBS gradient (over a 2 h period), where 50 000 cells were seeded upon our hydrogel sample placed within a transwell. After this time, the media in the well plates were removed, and replaced with FBS‐free media. In previous studies, we observed that while chemotactic gradients enhanced overall cell migration, they did not qualitatively alter the effect of confinement on migration, where the deformability of the cell nucleus emerged as a rate‐limiting step.^[^ ^42^, ^43^ ^]^ Thus, we anticipate that the presence of an FBS gradient greater than 10% would increase the overall number of cells that enter the scaffold, but not their relative distribution.

Microscopy was then used to observe cell migration at different locations in and on the hydrogel, i.e., on the surface, in channel intersections and in channels, as a function of hydrogel microarchitecture and stiffness.

**Figure 3:**
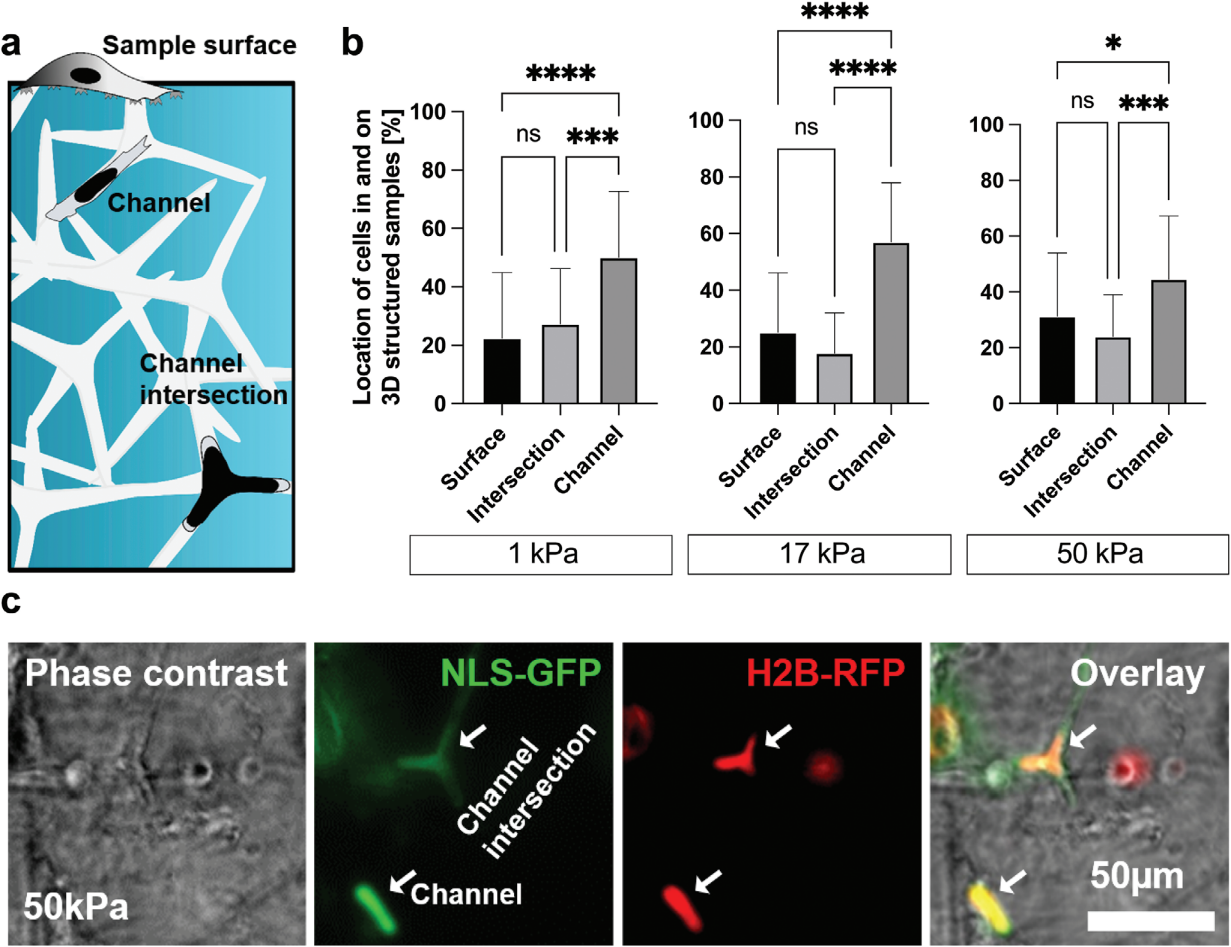
Location of cells in and on 3D structured samples after 5 d of incubation a) schematic of cell position within or on pAAm hydrogels. b) Percentage mean cell location for 1, 17, and 50 kPa pAAm hydrogels with mean and standard deviation (cells analyzed: 369 cells for 1 kPa, 287 cells for 17 kPa, 304 cells for 50 kPa), *p* 0.1234 (ns), 0.0332 (*), 0.0021 (**), 0.0002 (***), < 0.0001(****), mean ± SD. c) Example of cell within a channel and cavity of the 50 kPa microstructured hydrogel, images from left to right show phase‐contrast in gray (left) and the nuclei in green (NLS‐GFP) and red (H2B‐RFP), as well as an overlay.

**Figure** **3** shows the total percentage of cells observed in each hydrogel stiffness, and their specific location (surface, intersection, or channel) as observed through confocal microscopy after 5 d of cell incubation. In addition to this, we show an example of cell location in channels and intersections for a 50 kPa hydrogel. Note, here we describe only the means of cell location, and give representative images of how cells were identified, using phase contrast, NLS‐GFP and H2B‐RFP fluorescence.

While polyacrylamide is biocompatible in long‐term experiments if properly cross‐linked,^[^ ^43^ ^]^ we have in a previous publication confirmed the biocompatibility of pAAm networks for mammalian cells over 48 h.^[^ ^34^ ^]^ Although we did not observe significant changes in cell behavior, the microchannel geometry may nonetheless influence the metabolism of the cells, e.g., due to changes in the diffusion of nutrients.^[^ ^44^ ^]^

As shown, cells that infiltrate the hydrogels at 1, 17, and 50 kPa stiffnesses, tend to migrate towards channels, 47, 55, and 41% respectively. Our hypothesis as to why this occurs is based on the concave and convex features that are present within the 3D hydrogel network (Figure 1b). According to other findings cells actively avoid concave features,^[^ ^45^ ^]^ which may account for the observations we see with the intersections, and how they are less dominated by the cells.^[^ ^30^, ^45^ ^]^ From our results, some subjectivity in cell location can be expected as it is hard to identify the precise and complete hydrogel microstructure through confocal imaging of cells within this structure.

The normalization of cells in the different locations of the hydrogel (Figure 3b) was carried out by relating the number of cells in one specific location to the total number of cells present in this hydrogel. The cells were mainly located in the channels, presumably because more channels than intersections are present in the hydrogel (see Supporting Information).

#### 3.2.2 Nuclear Envelope Ruptures Are Controlled by Hydrogel Stiffness

**Figure 4:**
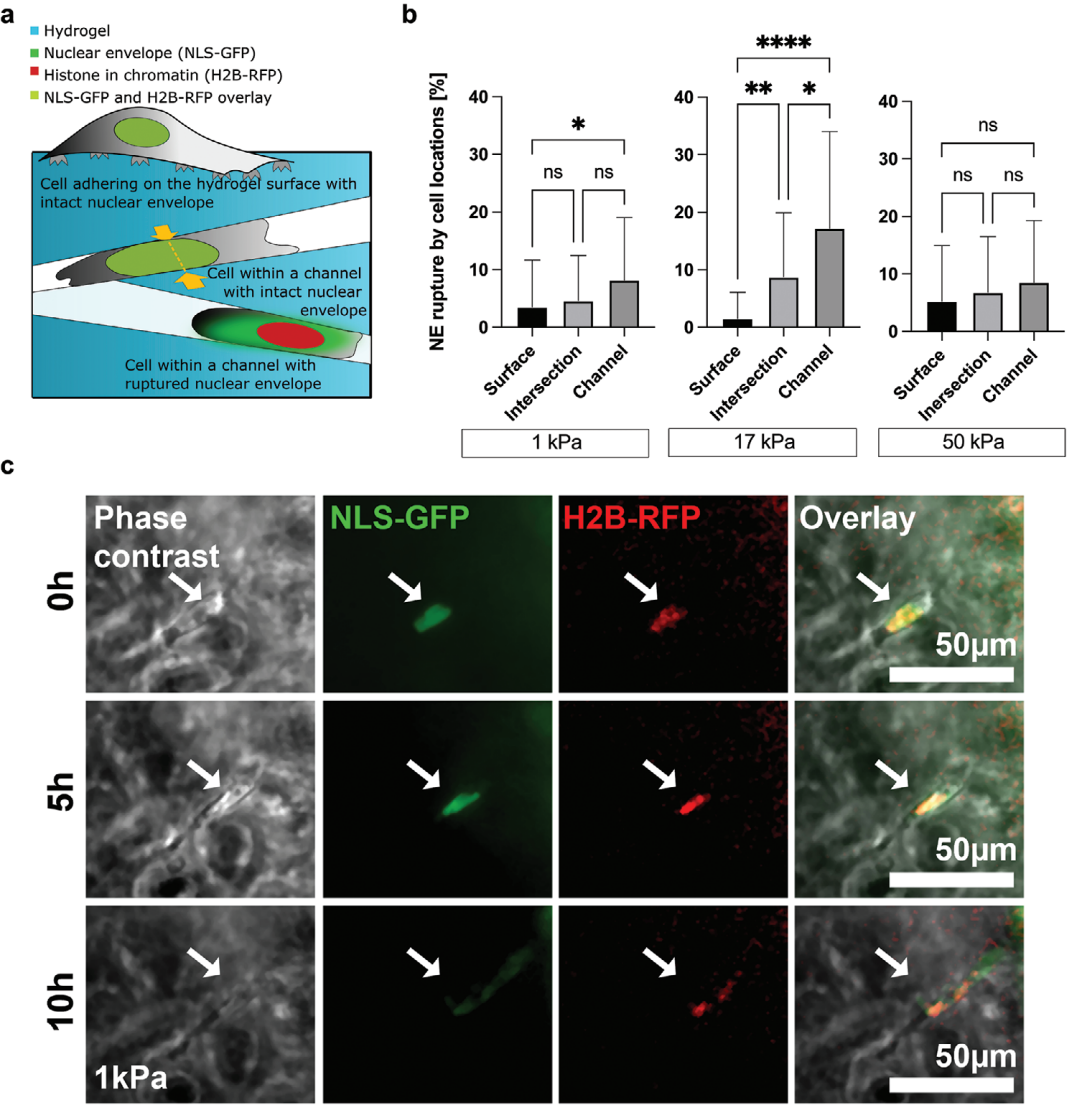
General features of nuclear rupture events. a) Schematic representation of cells with intact and ruptured nuclei within a microstructured hydrogel (blue) sample. The nucleus is labeled by the H2B‐RFP and the NLS‐GFP. In case of NE rupture, the NLS‐GFP can diffuse into the cytoplasm, resulting in reduced nuclear NLS‐GFP signal and in increased cytoplasmic NLS‐GFP signal. b) Percentage of NE rupture occurring in each area of the hydrogel as function of Young's modulus after 5 d incubation, as calculated through NE rupture events (in each section) divided by a total number of rupture events of counted (1 kPa, 67 NE rupture events observed from 369 cells, 17 kPa, 74 NE rupture events observed from 287 cells and 50 kPa, 63 NE rupture events observed from 304 cells), p 0.1234 (ns), 0.0332 (*), 0.0021 (**), 0.0002 (***), < 0.0001(****), mean ± SD. c) Example of NE rupture event over 10 hours for a 1 kPa hydrogel. Left: Image of phase contrast (gray) and the NLS‐GFP (green) and red (H2B‐RFP), as well as an overlay. The arrows point to the same cell, which exhibits NE rupture. The NE ruptures are visible by the loss of nuclear NLS‐GFP and an increase in cytoplasmic NLS‐GFP as shown from the two time‐points over 10 h.

Ruptures in the NE can compromise the integrity of the genomic material^[^ ^2^, ^46^ ^]^ and are an important readout for investigating the impact of mechanical force on the nucleus.^[^ ^10^, ^11^ ^]^ To determine NE rupture events in hydrogel channels as a function of hydrogel stiffness, we quantified NE rupture in the hydrogel channels using cells expressing H2B‐RFP and the NLS‐GFP rupture reporter. NE rupture is detectable by the NLS‐GFP spilling from the nucleus into the cytoplasm (**Figure** **4**a).

To investigate whether the matrix elasticity has an impact on the nuclear envelope ruptures, rupture events were collected over ten hours for each hydrogel and stiffness (Figure 4). Rupture events were not only calculated in relation to hydrogel stiffness, but in the specific locations within each gel itself. From the total cells counted for each stiffness, we observed 67 NE rupture events from 369 cells for our 1 kPa (18%), 74 NE rupture events observed from 287 cells within our 17 kPa (25%) and 63 NE rupture events observed from 304 cells in the 50 kPa hydrogels (20%) from the confocal images that were analyzed (*N* = 3, *n* = 10). From Figure 4b, we can further break this down to where the NE rupture events took place within the hydrogels. In general, we observed NE rupture events occurring most prominently in the channels. This can be attributed due to cell population as derived from Figure 3b. We also hypothesize that the NE rupture events occur more frequently in these channels due to the relative pressure associated upon travel through narrow constrictions.

The microchannel network in our hydrogels is substantially different from pore structures in previous studies. Whereas collagen scaffolds provide fibrous networks that the cells squeeze through,^[^ ^47^ ^]^ studies that implement PDMS such as Denais et al. and Holle et al., mimic cell migration in stiff, short‐range constricted environments (20 to 150 µm in length).^[^ ^10^, ^11^ ^]^ In previous studies on NE ruptures, the main focus was on the impact of channel geometry on NE ruptures, here we demonstrate that the stiffness of the surrounding matrix can also modulate the amount of NE rupture events. Although our studies tested conditions of NE rupture under mechanically softer constrictions than those previously studied,^[^ ^10^, ^11^, ^14^ ^]^ we still detected a significant number of NE rupture events in the microchannels. Figure 4c shows a typical nuclear rupture event taking place in a 1 kPa hydrogel. Firstly, at 0 hours, the cell nucleus of interest, has a compact cylindrical shape which can be seen from both the phase contrast and NLS‐GFP fluorescent image. Upon migration within the channel, nuclear elongation is observed after 5 h. Upon further elongation of the nucleus, NE rupture is evident through the dispersion of the NLS‐GFP signal into the cytoplasm, which is observed at the 10 h mark.

**Figure 5:**
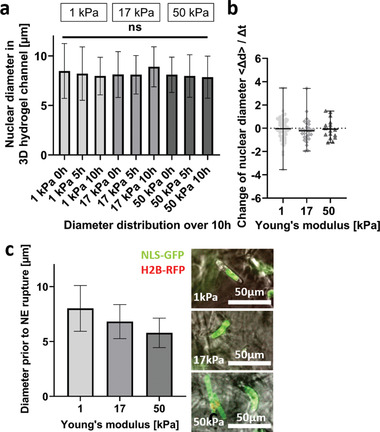
Nuclear diameter for cells in microchannels in hydrogels with 1, 17, and 50 kPa Young's modulus. a) Distribution of averaged nuclear diameters in microstructured hydrogels with Young's moduli of 1, 17, and 50 kPa. (study time: 10 h); cells analyzed for each stiffness and time point (stiffness: 0, 5, 10 h), 1 kPa: 66, 61, 53 cells, 17 kPa: 35, 30, 15 cells and 50 kPa: 40, 39, 35 cells, mean ± SD. b) Young's moduli dependent range of nuclei diameter change for individual cells within time intervals of 5 h. c) Nuclear diameter at or prior of the NE rupture. Error bars show means and standard deviations. *n* ≥ 10. Exemplary cell images directly before the rupture event or during the rupture event are shown. NLS‐GFP is used as a NE rupture reporter; H2B‐RFP labels chromatin within the nucleus.

As the above example illustrates, the nucleus adopts an ellipsoidal shape inside the hydrogel channels. When analyzed for all cells inside hydrogel channels, the minor axis of this ellipsoid shape was recorded at three‐time intervals, *t* = 0, *t* = 5, and *t* = 10 for multiple nuclei within each hydrogel stiffness. Independent of the hydrogel stiffness, the average (short‐axis) diameter of the nucleus was at around 8 µm (**Figure** **5**a). When analyzing nuclear dynamics, we found that change of the nuclear diameter over time (Δ*d*/Δ*t*) varied with hydrogel stiffness, where a larger distribution of changes was observed for soft hydrogels compared to the stiffer hydrogels (Figure 5b), indicating that the cells can probably deform the channels more easily at lower hydrogel stiffness.

To further investigate the interplay between hydrogel stiffness and nuclear dynamics, we measured the nuclear diameter of cells in microstructured hydrogels at or directly prior to the NE rupture event for hydrogels with different stiffness (Figure 5c). Interestingly, the prerupture diameter decreased with increasing hydrogel stiffness. We hypothesize that the reason for this finding is the low mechanical stiffness of the hydrogels, which enables cells inside the hydrogel to deform the channel walls as the stiff nucleus passes through the channels. In the softer 1 kPa hydrogels, the cell can deform and open up the channels significantly more before reaching a critical pressure on the nucleus that triggers NE rupture, whereas in the stiffer hydrogels, the cells can only achieve smaller deformations of the channel wall to reach the same critical pressure on the nucleus. These results provide first indications that rather than defined by the absolute channel diameter, it is the force exerted on the nucleus from the confining space that determines NE rupture.

#### 3.2.3 Positioning of the Nucleus Is Independent of Hydrogel Stiffness

**Figure 6:**
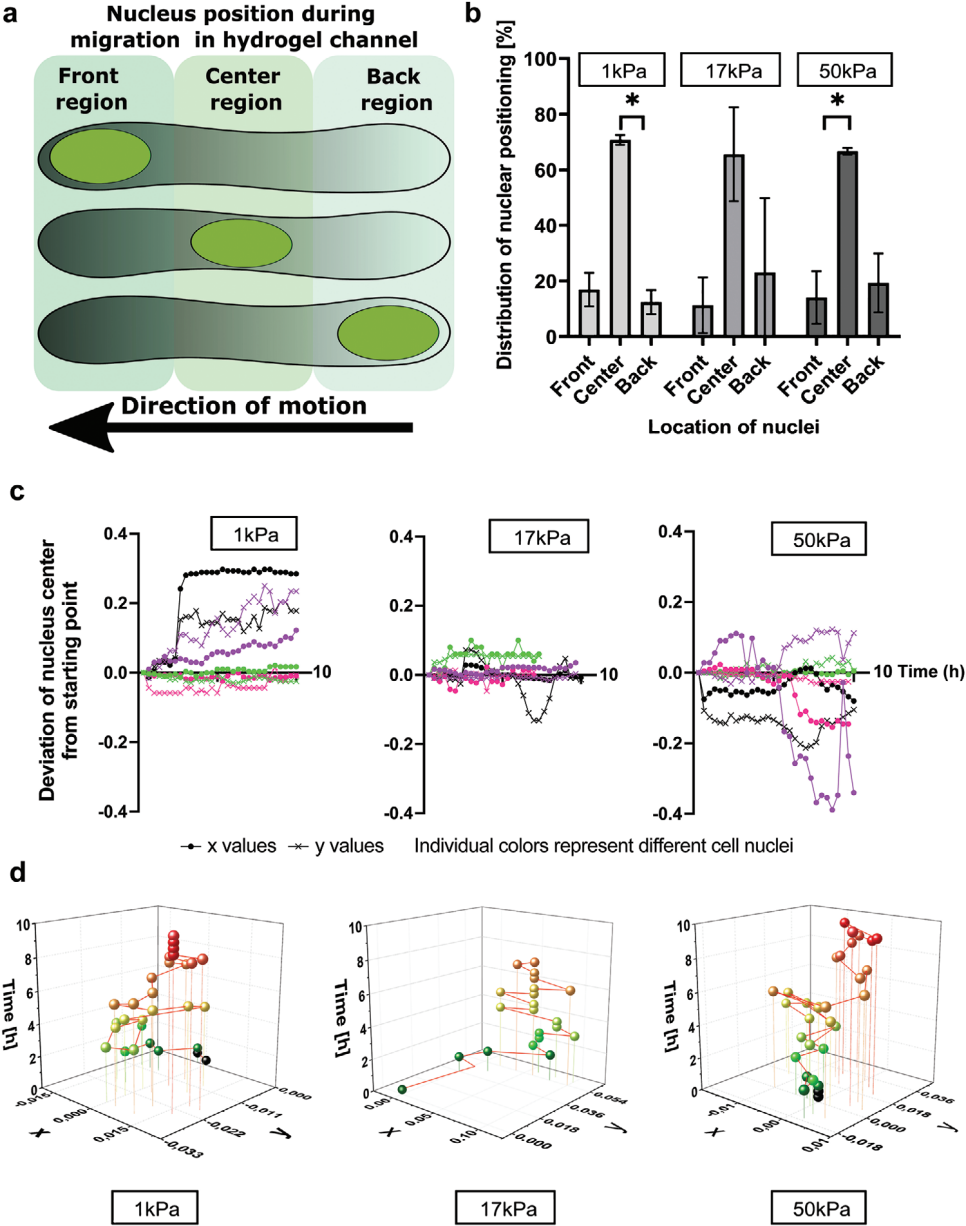
Nuclear positioning. a) Illustration of relative nuclear positions detected in the experiments. The color‐coded regions decide on the categorization: Front, center, and back region of the cell. b) Nucleus position as a function of hydrogel stiffness. (1 kPa: 153 cells, 17 kPa: 92 cells, 50 kPa: 93 cells), *p* 0.1234 (ns), 0.0332 (*), 0.0021 (**), 0.0002 (***), <0.0001(****), mean ± SD. c) Deviation of the nucleus center from its initial position over time displayed as normalized *x* and *y* coordinates. For each hydrogel stiffness, four exemplary cells represented with individual colors are depicted in a time period of 10 h. d) 3D representation of nuclear trajectories in 3D microchannels of the nucleus normalized to its initial position (green) during migration through microchannels (to red) in a 10 h time period for every 20 min. For each hydrogel stiffness, an exemplary trajectory is presented. All x and y values are given in arbitrary units.

Tracking the nucleus is a typical strategy to quantify cell movement in tiny constrictions,^[^ ^48^, ^49^, ^50^ ^]^ and nuclear positioning has a strong impact on cell migration and other cell functions.^[^ ^51^ ^]^ For each hydrogel, the position of the nucleus was observed upon cell migration in a single direction (within a channel) to specify how the cell nucleus changes its direction. Within an elongated cell, the nucleus was classified as being in one of three relative sections of the cell membrane (front, center, or back region) as shown in **Figure** **6**. The nucleus was predominantly located in the center of the cell body, regardless of hydrogel stiffness, whereas nuclear positioning in the front or back of the cell relative to the migration direction was relatively rare. HT1080 cells adhering on flat hydrogel surfaces have a nucleus positioned in the central part of the cell (Figure 2, Supporting Information).

**Figure 7:**
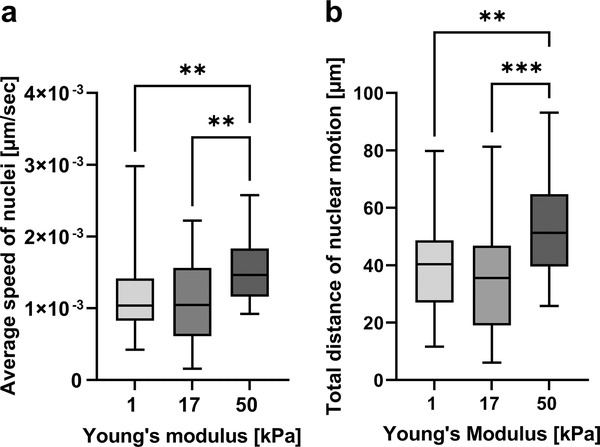
Analysis of the motion of cell nuclei. a) Average speed of nuclei in between image sequences of hydrogel stiffness [µm s^−1^]. b) Total distance of nuclear motion [µm]. Statistics shows box plots with mean ± SD and min to max values; *p* 0.1234 (ns), 0.0332 (*), 0.0021 (**), 0.0002 (***), < 0.0001(****).

Selected nuclei were then tracked by detecting their center of mass, as shown in Figure 6c,d. The trajectories of the nuclei from their starting point (at an arbitrary time point) are shown for four nuclei, for each hydrogel stiffness. The cell nuclei carry out zig‐zag‐like but continuous motion in one direction, which is particularly obvious from the 3D trajectories shown in Figure 6d. From the nuclear trajectories, we analyzed the speed of the nuclei (based on the nucleus center displacement over time) every 20 min over 10 h. The speed of the nuclei is a marker for cell speed and it increased significantly with increasing hydrogel stiffness **Figure** **7**. With the total distance of nuclear motion, the activity of the nuclei can be compared by the lengths of the tracks the nucleus moves over time. Significant differences are found for the total track lengths and thus for the cell activity in the stiffest hydrogel environment to the softer ones. These results are in agreement with previous migration studies on breast cancer cells, which also migrated fastest in stiff hydrogel channels.^[^ ^11^ ^]^

Whereas cell migration speed shows a biphasic dependence on cell adhesion strength on flat 2D substrates, this relationship does not necessarily hold true in confined 3D environments, where cells can also move in the absence of cell adhesion and migration is strongly dependent on the topology.^[^ ^52^ ^]^ Hence, we assume that the specific geometry provided by the channels in our hydrogels significantly alters cell migration.

## 4 Conclusion

Our study provides evidence that the mechanical properties of 3D hydrogels and microchannel networks strongly influence cell migration and nuclear rupture events, particularly when cells migrate through said channels. These findings highlight the importance of hydrogel stiffness, which is extremely relevant for biomedical applications, including regenerative medicine. A distinct advantage of our approach compared to other approaches is that our method for fabricating microchannels guarantees microchannel interconnectivity even at low channel volumes. This work enables for the first‐time studies on mammalian cell migration and nuclear envelope integrity in microchannel networks in synthetic 3D hydrogels without the need for complex 3D structuring methods, such as direct laser writing. Due to the interconnectivity of the channels, we were able to study how cells enter and migrate throughout the network. In particular, we were able to determine the effect of hydrogel stiffness and microarchitecture on cell invasion, nuclear dynamics, and NE rupture events. Therefore, our material strategy provides an important step towards the design of in vivo conditions in synthetic extracellular matrices by combining cell and nuclear envelope control through both 3D microchannel architecture and hydrogel stiffness.

## Conflict of Interest

The authors declare no conflict of interest.

## Supporting information

Supporting Information

## Data Availability

The data that support the findings of this study are available from the corresponding author upon reasonable request.
