# Supplementary material for: Tunable 3D Hydrogel Microchannel Networks to Study Confined Mammalian Cell Migration
Source: Adv Healthc Mater. 2021 Oct 20;10(23):2100625. doi: 10.1002/adhm.202100625 (PMC8743577; doi:10.1002/adhm.202100625)
Supplement: Supplementary file 1 — Supporting Information [file ADHM-10-2100625-s001.pdf]

WILEY-VCH

© 2021 Wiley-VCH GmbH

ADVANCED HEALTHCARE MATERIALS

Supporting Information

for *Adv. Healthcare Mater.,* DOI: 10.1002/adhm.202100625

Tunable Three-dimensional Hydrogel Microchannel Networks to Study Confined Mammalian Cell Migration

*Katharina Siemsen, Sunil Rajput, Florian Rasch, Fereydoon Taheri, Rainer Adelung, Jan Lammerding, Christine Selhuber-Unkel**

Supporting Information

Tunable three-dimensional hydrogel microchannel networks to study confined mammalian cell migration

*Katharina Siemsen, Sunil Rajput, Florian Rasch, Fereydoon Taheri, Rainer Adelung, Jan Lammerding, Christine Selhuber-Unkel**

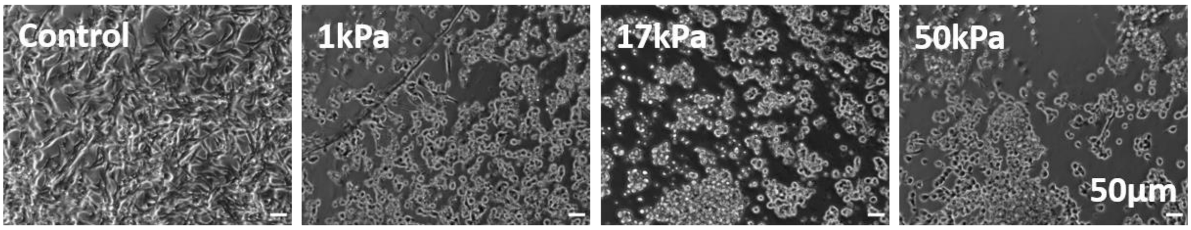

SI Figure 1. Phase contrast images showing both controls for cell seeding (HT1080) within a 24-well plate as well as 2D collagen I functionalized hydrogels (1, 17 and 50kPa) after 5 day incubation. Cells were treated with the same volume of medium and the 2D hydrogels were incubated into trans wells, similar to the 3D microstructured hydrogel. Scale: 50µm

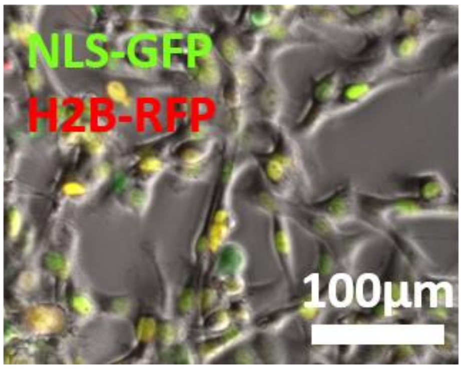

SI Figure 2. Merged phase contrast and fluorescent images show a control of cell seeding (HT1080) within a 24-well plate after 5 days of incubation. Cell nuclei are mainly located in the center of the adhering cells. Scale: 100µm

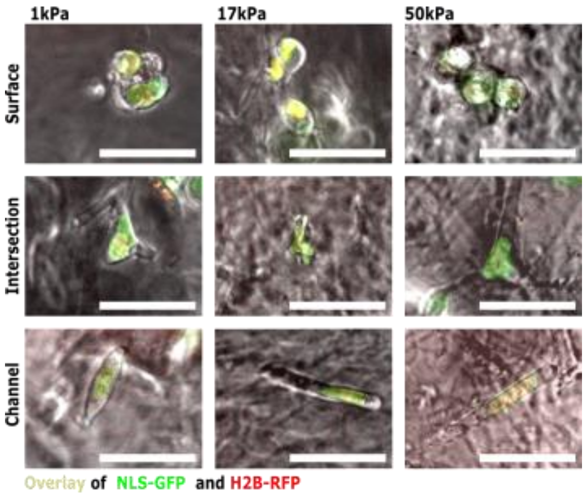

SI Figure 3. Systematic image about cell location and hydrogel stiffness. Image represent overlays of phase contrast and NLS-GFP (green) and H2B-RFP (red) channels. Scale: 50µm

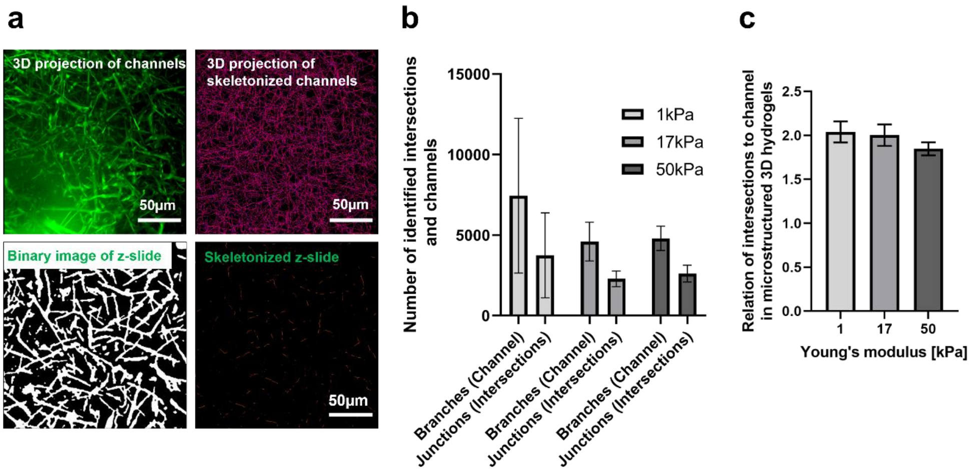

SI Figure 4. Analysis of channel architecture based on fluorescent 3D image stacks. a) 3D projections of channels, skeletonized channels, the respective binary image and the skeletonized z-slide, derived from hydrogel volumes of 3.5 × 10^6 µm^3 (41 z-slices, distance 1.99µm). Scale bar 50µm. Green: FITC-dextran. b) Number of intersections and channels identified as a function of Young's modulus. The bar graph shows mean ± standard deviation. c) Ratio of the number of intersections to channels in the microstructured hydrogels, as a function of the hydrogels' Young's modulus. Graphs shows mean ± standard deviation. Differences were not statistically significant.
